# Supplementary figures and images for: AEGIS: Individual-based modeling of life history evolution
Source: PLoS Comput Biol. 2026 Mar 26;22(3):e1014109. doi: 10.1371/journal.pcbi.1014109 (PMC13020811; doi:10.1371/journal.pcbi.1014109)

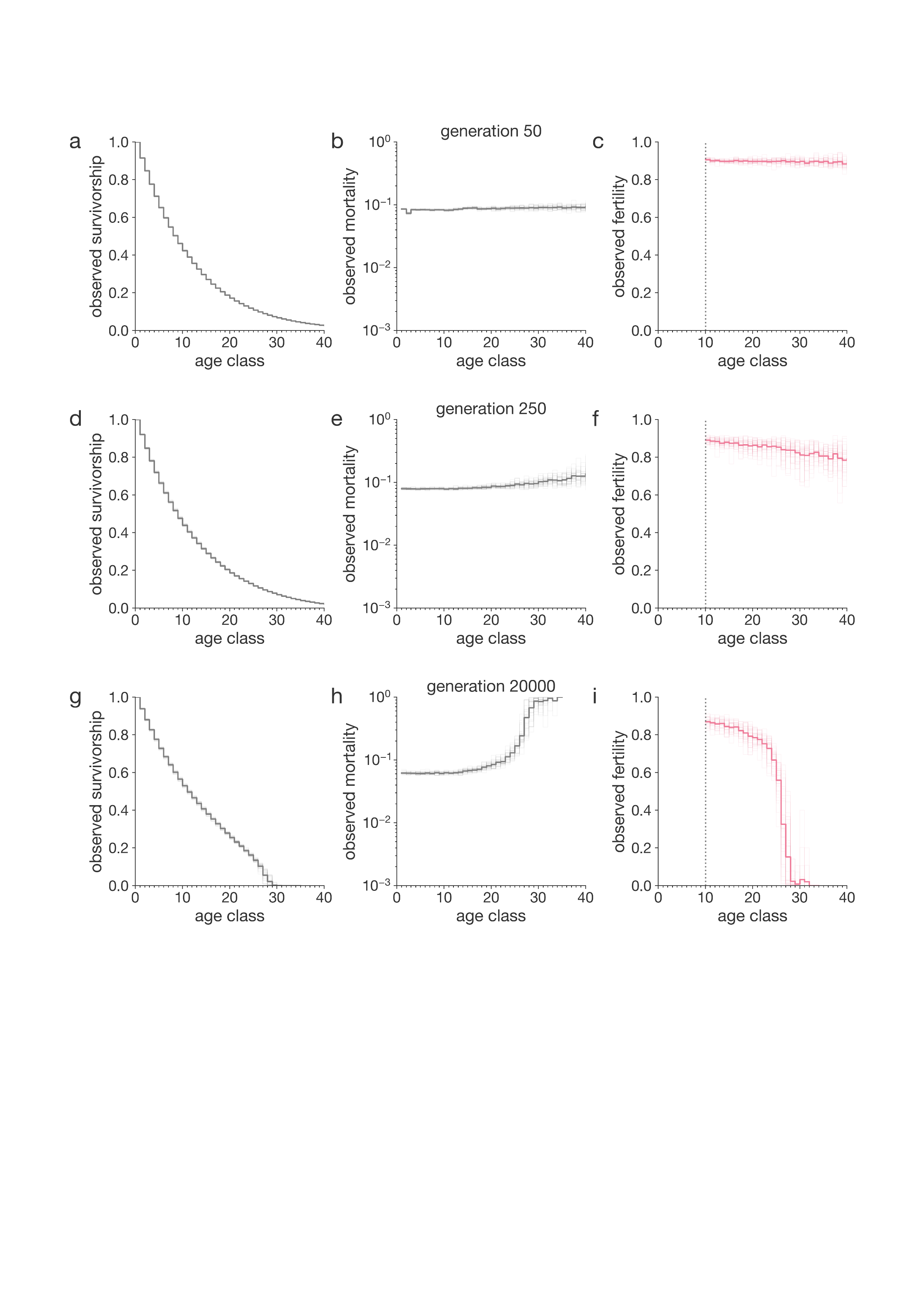

Supplement: S1 Fig — Average population-level observed survivorship, mortality and fertility curves at generation 50 (a-c), 250 (d-f) and 20000 (g-i). At generation 50, no age-dependent pattern in mortality nor fertility is discernible. At generation 250, observed mortality increases with age, while observed fertility decreases with age. At generation 20000, same trends are visible, but they are more intense. Furthermore, they are indistinguishable from phenotypes at generation 200000 (Fig 4a-4c). (TIF) [file pcbi.1014109.s001.tif]

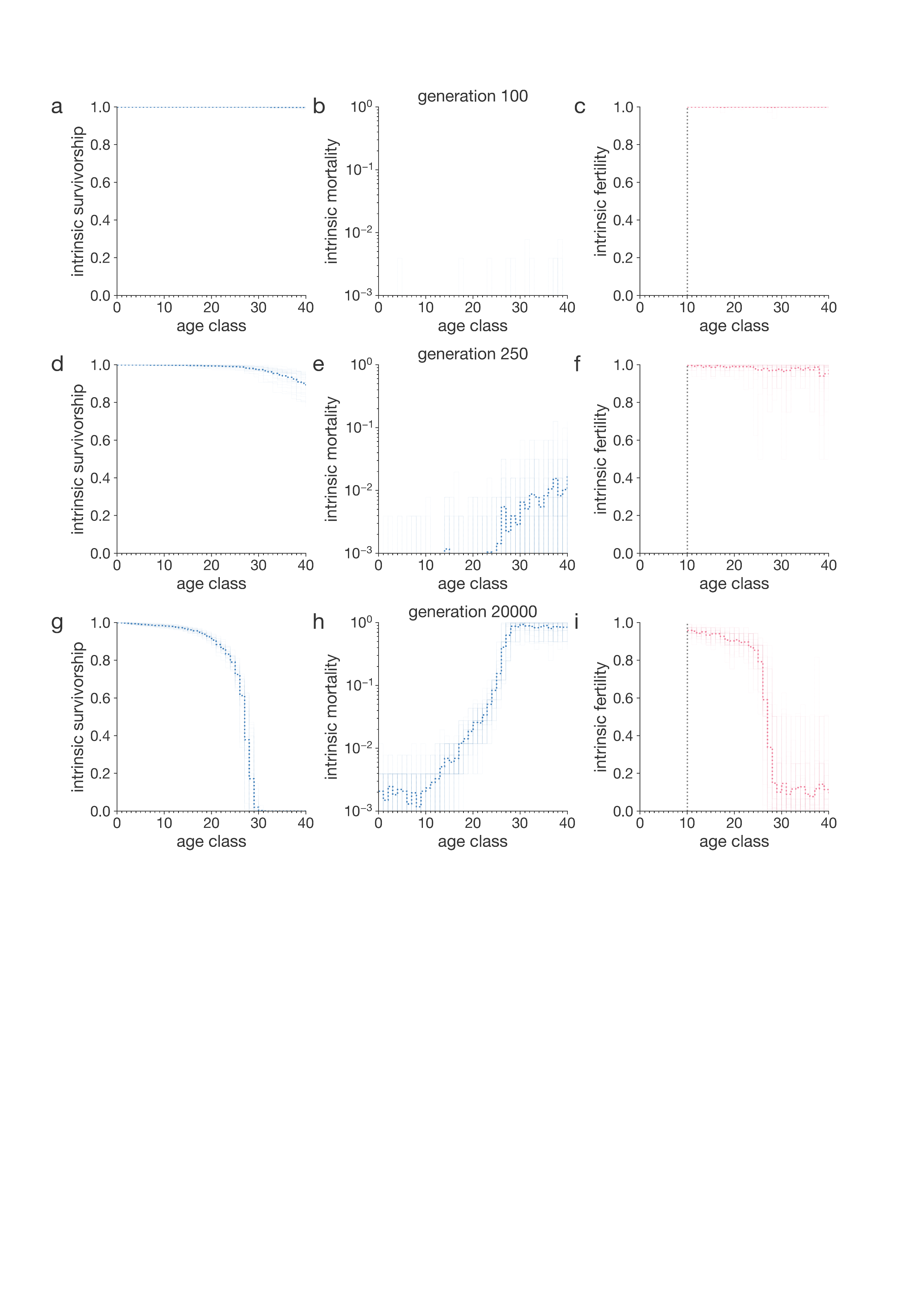

Supplement: S2 Fig — Average population-level intrinsic survivorship, mortality and fertility curves at generation 50 (a-c), 250 (d-f) and 20000 (g-i). At generation 50, no age-dependent pattern in fertility is discernible and mortality is extremely low. At generation 250, observed mortality increases with age, while observed fertility weakly decreases with age. At generation 20000, age-specific trends emerge which are also much more intense. Furthermore, they are indistinguishable from phenotypes at generation 200000 (Fig 4d-f). (TIF) [file pcbi.1014109.s002.tif]

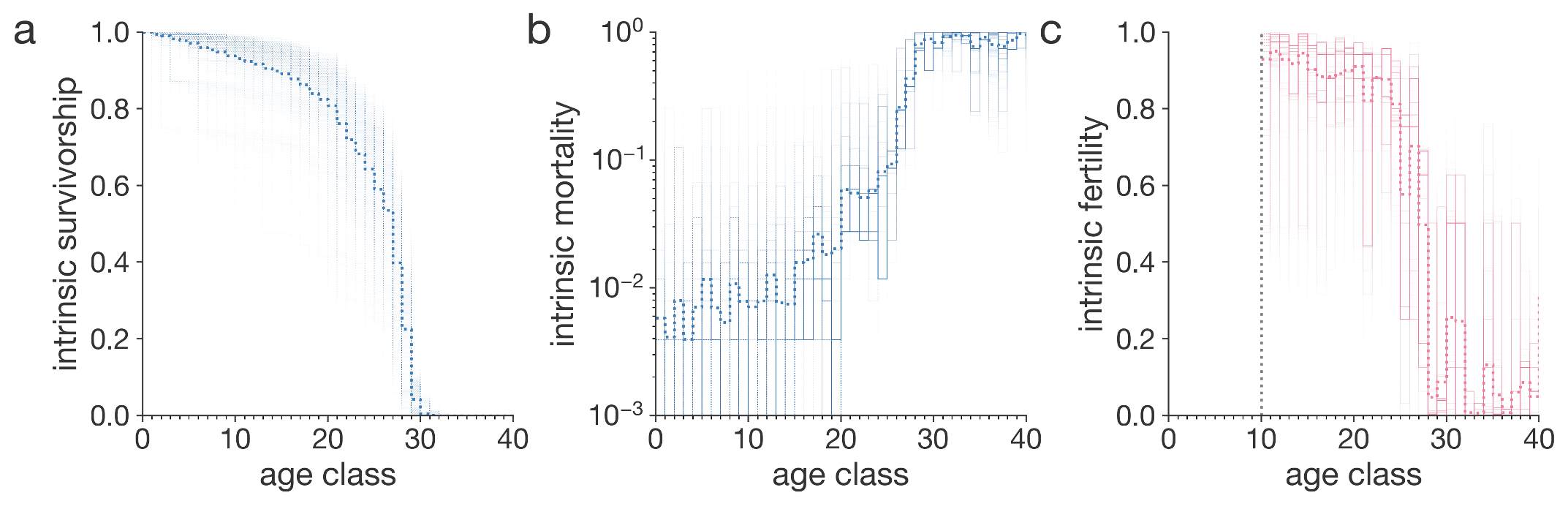

Supplement: S3 Fig — The life history curves shown are based on 100 simulations which ran for three million steps. Thick curves are population averages. Thin lines are individual-specific traits. Plotted traits are survivorship (a), mortality (b) and fertility (c). Survivorship is commonly understood as an observed trait of a cohort rather than an intrinsic trait of an individual; however, we include it since it is visually informative. The technical interpretation of individual intrinsic survivorship is the survivorship of a hypothetical cohort of genetically identical individuals that die only due to intrinsic causes. (TIF) [file pcbi.1014109.s003.tif]

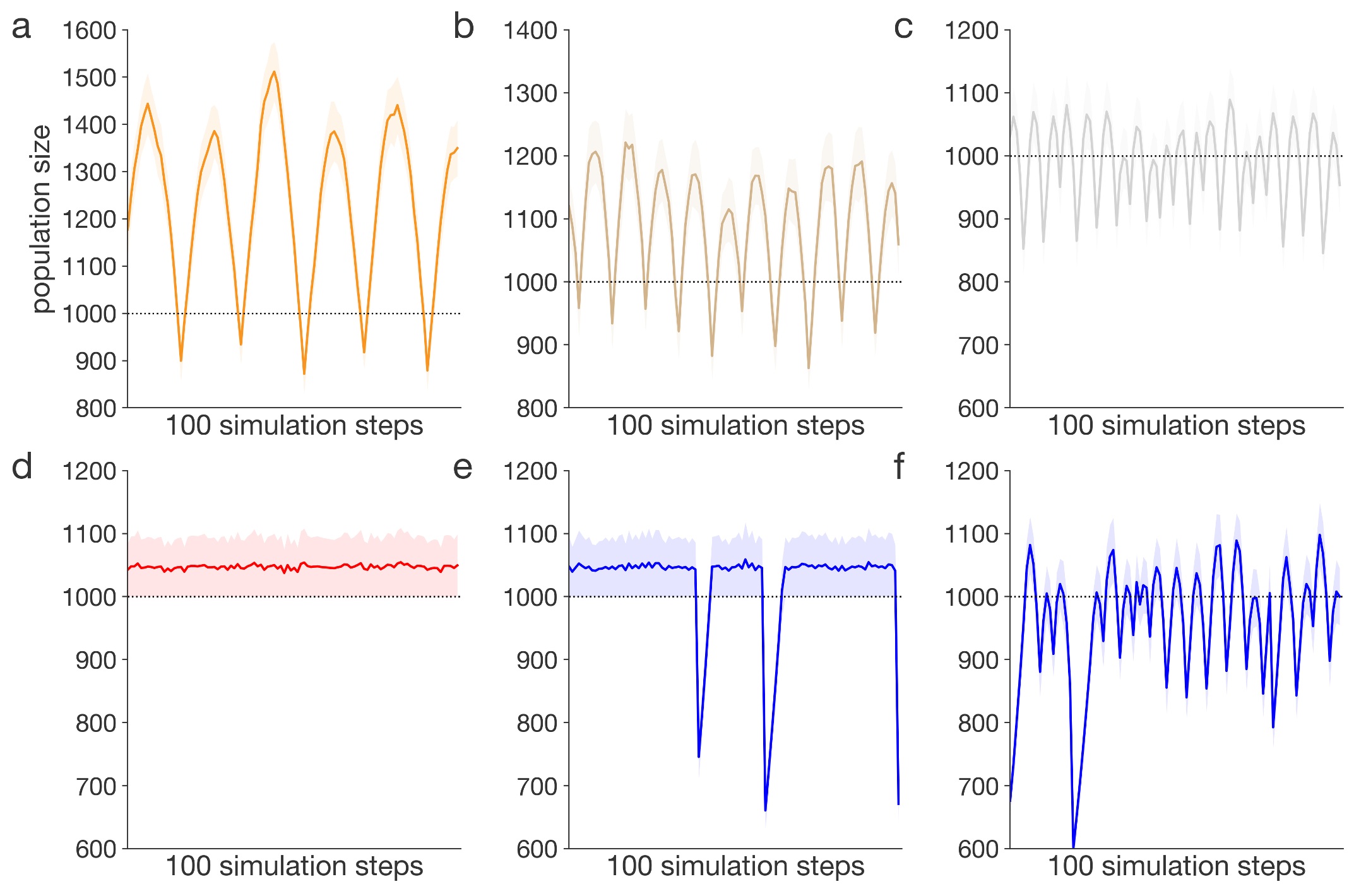

Supplement: S4 Fig — Six simulations with six different extrinsic mortality regimes. Carrying capacity in all simulations is 1000. The shaded areas depict the population size during a single step; the bold lines are averages. The first three panels (a-c) show populations that exhibit different sensitivities to starvation, i.e., different increases in mortality under lack of resources. Population a is most starvation-resilient, while the population c is least starvation-resilient. Populations d responds to overshooting precisely, thus do not dip below the carrying capacity. Populations e and f experience bursts of periodic abiotic mortality (every 25 steps) of random magnitude. Population e responds to overcrowding precisely, while population f suffers under overcorrection. (TIF) [file pcbi.1014109.s004.tif]

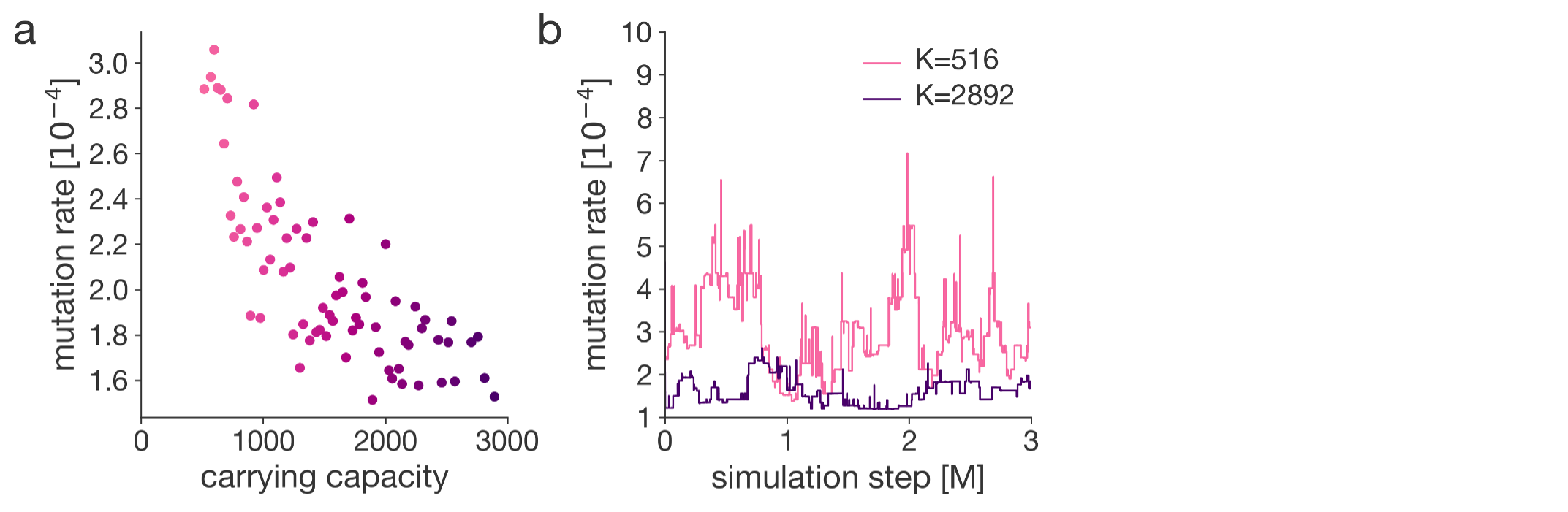

Supplement: S5 Fig — The evolved mutation rates shown are based on 100 simulations which we ran for three million steps. Environment is stable; i.e., fitness of each phenotype does not depend on simulation time. a, Population- and time-averaged mutation rate for populations evolving under different sizes. b, Population-averaged mutation rate over the simulation time. The darker line represents the average mutation rate of the largest population (K = 2892), the lighter line of the smallest population that did not go extinct (K = 516). Populations are reproducing asexually. (TIF) [file pcbi.1014109.s005.tif]

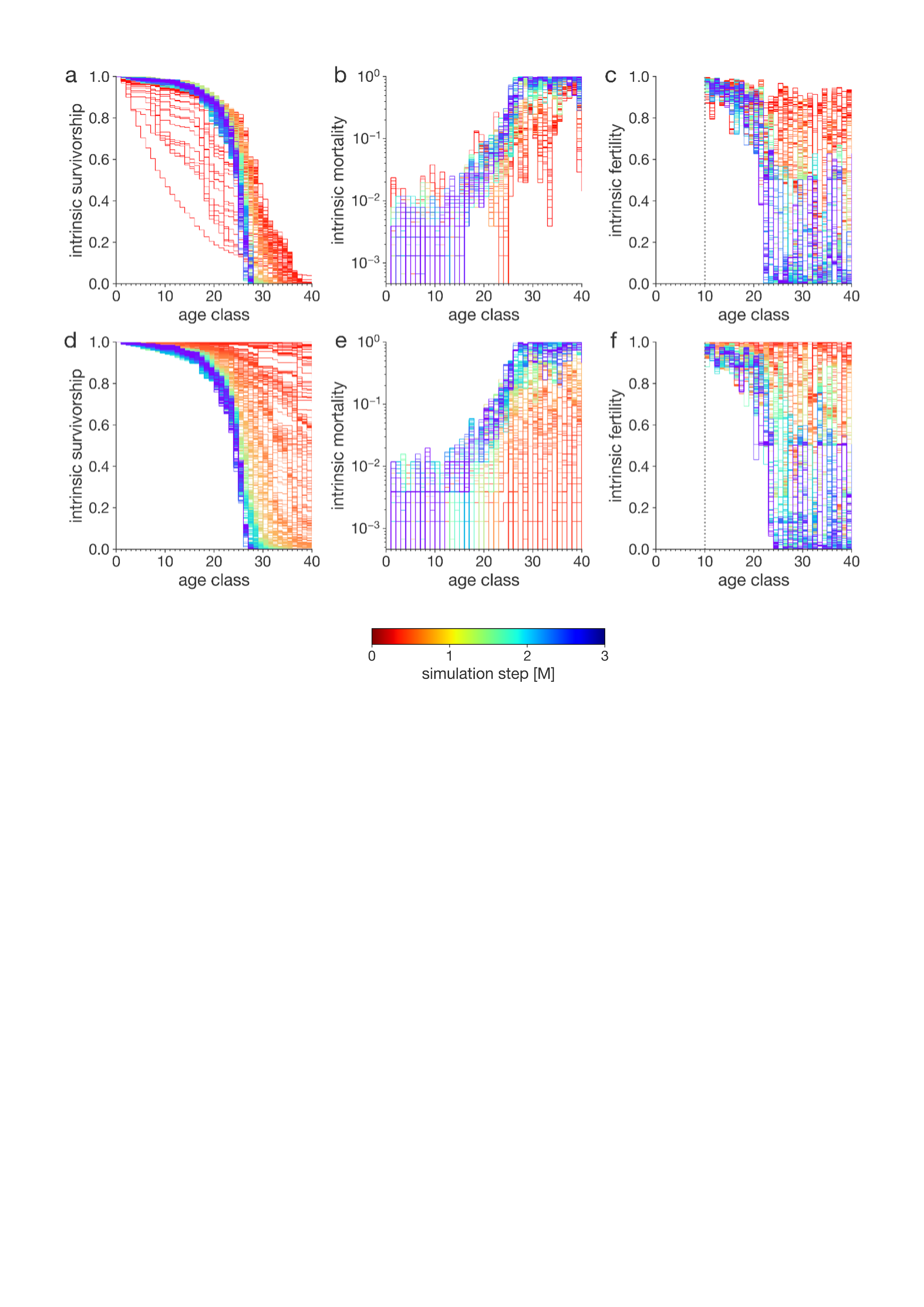

Supplement: S6 Fig — The life history curves shown are based on 100 simulations we ran for three million steps. Red represents phenotypes recorded early in the simulation, violet late. For simulations depicted in panels a and b, mortality was initialized as high, while in panels d and e, it was initialized as low. Evolved states (in violet) do not differ significantly. For simulations depicted in panel c, fertility was initialized as low, while in panel f it was initialized as high. Evolved fertilities (in violet) do not differ significantly. (TIF) [file pcbi.1014109.s006.tif]
