# Supplementary material for: AEGIS: Individual-based modeling of life history evolution
Source: PLoS Comput Biol. 2026 Mar 26;22(3):e1014109. doi: 10.1371/journal.pcbi.1014109 (PMC13020811; doi:10.1371/journal.pcbi.1014109)
Supplement: S1 File — (DOCX) [file pcbi.1014109.s007.docx]

**Supplementary Information**

## Parameters and their explanations

The AEGIS model comprises several distinct modules, each simulating specific biological processes; e.g., an infection module simulating disease dynamics, and a predation module simulating predator-prey interactions. Each module is structured with both fixed and modifiable components. The fixed logic refers to hard-coded elements that cannot be altered by the user. For instance, within the infection module, individuals are constrained to two health states: *infected* or *healthy*. However, modifiable logic allows for user-defined adjustments to certain parameters. In the infection module, for example, the fatality rate (the probability of death per simulation step given an infection) can be customized via input parameters.

A list of parameters, including brief explanations, default and allowed values, are all available on GitHub (<https://github.com/valenzano-lab/aegis/blob/v2/src/aegis/documentation/dynamic/default_parameters.md>). To fully understand the role and behavior of each parameter, users are encouraged to consult the module descriptions, which detail both the fixed and modifiable logic of each module which is important for understanding how a parameter contributes to the overall module logic. Module descriptions are within the AEGIS GUI but also in a text format on GitHub (<https://github.com/valenzano-lab/aegis/blob/v2/src/aegis/documentation/dynamic/submodel_specifications.md>).

## Default parameter values

Default parameter values are provided at <https://github.com/valenzano-lab/aegis/blob/v2/src/aegis/documentation/dynamic/default_parameters.md>. Here, we explain the rationale behind these defaults and their implications for simulations. Although AEGIS uses default parameters modeled on key human life history traits—chosen because they are well-studied and of broad interest—several important deviations were made. These deviations were necessary to enhance computational efficiency, ensure broad applicability across taxa, and simplify traits that are either highly complex or difficult to generalize.

Under default parameterization, reproduction is modeled as panmictic, without incorporating sexual selection, mating systems, monogamy, or menopause, and gamete aging is not considered. Epigenetic processes are excluded, and the genome size is reduced, though germline mutation rates remain consistent with empirical levels in humans; also, they do not evolve. The model assumes that genetic variants exhibit age-specific and trait-specific effects. Starvation dynamics are also simplified, with susceptibility and resource consumption uniform across individuals and ages.

To accurately model human populations, additional features beyond the scope of AEGIS might be beneficial. These include socio-cultural factors, such as intergenerational transfers, as well as biological and ecological processes like migration, gene flow, admixture, and the impact of medical or technological interventions.

## Termination condition

When investigating long-term equilibrium in evolved life history traits, it is essential to confirm whether the simulation has indeed reached an equilibrium state. Longitudinal comparisons of life history traits can aid in this process, as a stable trait over time—despite some fluctuations—should not exhibit any consistent trend, such as an increase or decrease over evolutionary time. Here, we present an alternative approach: running two simulations with differently initialized populations—one with low initial mortality and the other with high initial mortality. The hypothesis is that if the equilibrium value lies between these initial extremes, both simulations will converge on an intermediate value. Once convergence occurs, it can be inferred that the system has reached equilibrium.

This approach is illustrated in **S6 Fig**. S6 Fig is intended as a technical convergence test rather than a biological experiment: it assesses whether AEGIS converges to the same asymptotic life-history traits when initialized from distinct survival and reproductive states. Convergence in this setting indicates that the equilibrium is intrinsic to the model dynamics and not an artifact of initial conditions. After running AEGIS for 200,000 generations, both the high-mortality ancestral population (**S6b Fig**) and the low-mortality ancestral population (**S6e Fig**) converge on nearly identical mortality curves, as shown in the survivorship curves (**S6a** and S6**d Fig**). We conducted analogous experiments with differently initialized fertility conditions and found that fertility curves converge regardless of the initial low (**S6c Fig**) or high fertility states (**S6f Fig**). The convergence of mortality and fertility traits strongly suggests that the simulations have reached an equilibrium state, free from the influence of initial conditions.

It is important to note that this method is ineffective if the equilibrium value lies outside the initial extremes—either higher than both initial values or lower than both. In such cases, both simulations will display the same directional trend, with one simply lagging behind the other.

## Extensibility of AEGIS

Species evolve under diverse conditions in the wild and in laboratories [59], necessitating significant customization of our evolutionary models. Individual-based models, with their modular architecture, are particularly well-suited as community tools for simulating complex, dynamic, and stochastic processes due to their inherent flexibility. As an example, we implemented evolvable germline mutation rate, as a separate quantitative trait with a genetic basis, because it is not a life history trait itself, but it is highly variable across species [60] and it coevolves with life history traits [61-63].

We aimed to recapitulate the drift-barrier hypothesis of mutation rate evolution [40] by running one hundred simulations of sexually reproducing populations under carrying capacities varying from 300 to 3000 individuals. We observe that the mutation rate equilibrates at a level lower than what is expected by neutral evolution alone (**S5 Fig**). Furthermore, we find that larger populations are able to evolve lower germline mutation rates; i.e. there is an inverse relationship between effective population size and evolvable trait performance – a finding that corroborates the drift-barrier hypothesis and suggests that AEGIS can capture more general mechanics of evolution.

## Notes on model design

Here we list and discuss technical design choices that might be useful to consider when designing and interpreting simulations in AEGIS.

*Age and genetics dependence of sources of mortality*

AEGIS features five distinct sources of mortality, all of which could theoretically be modulated by the age and genetics of the affected individual. Although implementing age and genetic dependence for all five mortality sources is compatible with the current AEGIS codebase, the current version supports age dependence for starvation mortality and intrinsic mortality, as well as genetic dependence for intrinsic mortality.

*Juvenile mortality*

In most species, juvenile individuals experience mortality rates higher than the baseline due to increased susceptibility to environmental challenges. Under default parameter settings in our simulations, populations do not evolve juvenile mortality rates exceeding the baseline pre-maturity mortality. This suggests that additional conditions, such as heightened juvenile vulnerability, increased exposure to hazards, or trade-offs with other life-history traits, may be required for such an evolution. Although unrealistic for empirical settings, this setup serves as a neutral modeling baseline, since there is a multitude of potential intrinsic constraints and trade-offs, which could be implemented in various ways, none of which is inherently more valid than others. If relevant to specific simulations, these parameters should be tailored by the user.

Another factor potentially contributing to observed patterns is the age-specificity of mutations, wherein mutations affect individuals at single specific life stages. In contrast, when mutations persist across the lifespan, overall population mortality declines, as individuals with higher mutation loads and elevated mortality rates die off, leaving longer-lived, lower-mortality individuals. This process may underlie the age-dependent decline in mortality rates at the population level, and a higher-than-baseline juvenile mortality.

*Mutation rate*

Mutations occur only in the germline, there are no somatic mutations. Mutation rate determines the independent probability of each genomic bit to flip its binary state (from 0 to 1 or from 1 to 0). Backmutations are possible. The rate can be defined or be let to evolve. For a default genome size of thousand sites, and a default mutation rate of 0.001, each offspring will carry, on average, one new mutation. This contrasts with human 6.4 billion sites, and a mutation rate of 0.5 in a billion which results in tens of mutations per generation; many of which are silent or otherwise neutral.

*Simulation time steps*

AEGIS does not set a fixed interpretation of how much time each step lasts. The interpretation of a step is left to the modeler – it can be a day, a month, a year, or any other amount of time. Since the simulation progresses in steps, we usually do not use the term *age* but the term *age class* to highlight that it covers an age interval.

For some use cases, it is sufficient to measure age in generic time units but if the modeler wants to model a particular species, they should decide on the unit to ensure consistency when customizing other parameters. For example, when modeling humans, if one decides on one year as the time unit, one might set age limit to 100 and maturation age to 18. Alternatively, if one decade were the time unit, the modeler would set the parameters to 10 and 2, respectively. In the default case, the parameters are set to 50 and 10 (and the hidden choice of time unit is 2 years). If the modeler does not deliberate the time unit, the parameters might be expressed in different implicit time units, leading to an incoherent and, thus, non-representative model.

The choice of a time unit presents a tradeoff – short time units mean increased resolution (more granular life history records) but higher computational costs. A practical choice of the time unit will result in age limit to be set to a value between 20 and 50. This provides good resolution all while keeping computational costs tractable.

*Age limit*

As a non-parametric, numerical model, AEGIS is able to simulate life history traits of arbitrary shapes, unrestrained to functional forms, such as Gompertz mortality. The tradeoff of such a design is a relatively higher memory load. To keep the memory load finite and tractable, the modeler must specify the *age limit* ­– the highest number of steps that an individual can remain in the simulation. If an individual reaches the age limit, they will be removed even if their intrinsic life history traits and the environment would allow for their survival. This constitutes an artifact, a technical death, which should be avoided. The modeler should thus choose an *age limit* that is high enough that no individual (in an evolved population) reaches it but low enough to keep the simulation tractable and fast.

Note that the maximum lifespan and exceptional lifespan of the evolved population will be different numbers, lower than the parameter itself. The alternative name for the *age limit* parameter under the used software version is *maximum lifespan*.

*Bit genomes*

AEGIS simulates genomes as sequences of bits (**bit genomes**). They differ from real genomes in a couple of important ways – they provide no reference to specific real genes, and they encode no structural nor molecular mechanistic information (thus concepts such as splicing, intron/exon, promotor, transcription, translation, expression, etc. do not directly apply to them). However, they present advantages of being fully transparent – sequences are complete and fully phenotypically characterized.

Different theoretical approaches model genomes in distinct ways [64], each with its own advantages. The method implemented in AEGIS is optimized for high performance, enabling simultaneous modeling of multiple intrinsic processes and environmental factors.

*Age-specific patterns of mutational effects*

Phenotypic effects of most genetic variants of non-model species remain elusive; even the function of many affected genes themselves. Still less is known about the age dependency of the effects of mutations. However, it is expected that most genes and genetic variation affects individuals throughout most of their lives. In AEGIS, under default parameters, all variants have age-specific effects – they modify a life history trait at a specific age and are functionally silent at all other ages. Alternatively, variants could affect all ages (customizable by the user), however this modeling choice will constrain the space of evolvable phenotypes. For example, if variants affect all ages uniformly, then all evolvable life history traits will have flat age curves; or, if all variants had exponential age dependencies, all evolvable life history traits would change exponentially. AEGIS is designed to be species-agnostic per default, so such assumptions cannot be made as life history traits have diverse age dependencies across species [46]. Age-specific effects of variants, though unrealistic, allow the population to evolve age dependencies for life history traits of arbitrary shape.

*Scaling of mutational effects*

Additionally, to being age-specific, variants in AEGIS have additive (not multiplicative) effects in the sense that they modify a life history trait at a specific age by an absolute amount (e.g. reduce survival by 10 percentage points, e.g. from 50% to 60%; rather than by 10%, e.g. from 50% to 45%).

Survival, reproduction and per-locus mutation rates are limited to a range between 0 and 100%. Thus, mutations that push life history traits beyond the 0–100% range are effectively silent.

*Customizable genetic basis of aging*

Theories of *mutation accumulation* (MA)*, antagonistic pleiotropy* (AP) and *disposable soma* (DS) are considered classical evolutionary theories of aging which hypothesize different genetic architectures of aging. While the *mutation accumulation* theory postulates that aging is shaped by variants with detrimental late-life effects, the *antagonistic pleiotropy* theory suggests that the relevant aging variants have additional beneficial early-life effects, thus being pleiotropic. The *disposable soma* theory proposes antagonistic pleiotropic effects as well but across survival and reproduction. It is still unknown what their respective role is in shaping the aging phenotypes and life history phenotypes. Our implementation of the pseudogenome enables flexible simulation of diverse genetic architectures, including pleiotropic effects across ages and traits, thereby facilitating exploration of key genetic theories of aging. Moreover, the framework accommodates the simulation of established conceptual models, such as the Penna model [29].

Though mortality increases exponentially in many species, many age-related diseases exhibit non-linear age trajectories [65-67].

Under default settings, genetic variants are age-specific, affecting a single age only. However, AEGIS can simulate variants with linear or non-linear age trajectories, providing a means to investigate how such variants contribute to observed life history traits. The platform also allows exploration of their evolutionary dynamics, offering insights into the unresolved question of whether aging-associated variants are shared across individuals or largely private [68]. The degree of sharedness has important implications for the potential to intervene in the aging process at the population level.

*Modifying vs composite genetic basis of aging*

Genetic basis of life history traits in AEGIS can be modeled using two approaches that we can call *modifying* and *composite*.

The *modifying genetic basis* captures a common conceptual approach for modelling how genetic variants influence life history traits—each variant alters a trait (or multiple traits) relative to an inherited baseline. Mutation effect sizes are drawn from a *Beta(1,3)* distribution and scaled by a user-defined factor, resulting in most mutations having near-zero effects and none exceeding the specified maximum. This approach is particularly useful for modelling pleiotropy, for intuitive interpretation of the genetic basis of traits, and when reliable baseline life history parameters are available.

When such conditions are not met and a simpler, faster model is preferred, users can instead employ the *composite genetic basis*. In this framework, each locus in the 1-bit state increases a life history trait by a predefined value. When all loci are in the 1-bit state, survival and fertility reach 100% across all ages; conversely, when all loci are in the 0-bit state, both traits fall to 0%. Intermediate configurations yield intermediate life history rates. Users can also define different effect size distributions—for example, equal effect sizes across loci, or architectures with a few loci of large effect and many of smaller effect.

**Supplementary Methods**

## Technical requirements and installation

AEGIS is an open-source simulation tool requiring Python3 for operation. The software can be installed locally via the Python package manager pip (`pip install aegis-sim`) or accessed via a web server. For local installations, simulation speed is primarily constrained by CPU performance, as GPU acceleration is not utilized. The available system memory determines the maximum allowable population size and genome complexity within simulations. AEGIS outputs data in non-proprietary formats, predominantly using standard text files (.csv, .json), with certain outputs stored in binary formats such as Feather (https://github.com/wesm/feather) or Python pickle (<https://docs.python.org/3/library/pickle.html>).

## Reproducibility

Data were generated using the Python package **aegis-sim** (v2.2–3 on GitHub at https://github.com/valenzano-lab/aegis). To reproduce results, generate configuration files by running the *config.ipynb* script and run simulations as described in the script (by using the `aegis sim -o {config_file}.yml` command on all generated configuration files). To make plots using the generated data, run the *plot.ipynb* script.
