# Supplementary material for: AEGIS: Individual-based modeling of life history evolution
Source: PLoS Comput Biol. 2026 Mar 26;22(3):e1014109. doi: 10.1371/journal.pcbi.1014109 (PMC13020811; doi:10.1371/journal.pcbi.1014109)
Supplement: Attachment — Submitted filename: 2026.01.29_Response-to-reviewers_DRV.docx [file pcbi.1014109.s009.docx]

**Response to Reviewers:**

***Reviewer #1:*** *Bagic et al., propose a software suite for simulating the evolution of life history traits.*

*Their proposed solution surely makes it relatively easy to run simulations for these “ageing evolution” models. It is very easy to use through the web server as well as either through the pip install that went smoothly. In addition, the visual representations are easily understandable.*

*As mentioned by the authors lines 73-75, IBM are bottom-up models that allow emergent behaviors instead of needing to implement specific rules. However, numerous assumptions made for the implementation of the model (i.e. the sequence of events affecting an individual) seem to create unevaluated implicit constraints.*

*The implementation of the calculations methods themselves seem to be based on “An In Silico Model to Simulate the Evolution of Biological Aging”, double posted on arxiv and biorxiv for which I could not find a peer-reviewed version. The original paper does not seem to be comparing the proposed model with existing ones making it hard to understand what the conceptual or technical advances come along with this new implementation. For example, how does the current implementation of the code execution time compare with existing methods.*

We appreciate the reviewer’s insightful comment.

Indeed, a multitude of improvements have been made between the previous and current models, including feature expansion (e.g. implementation of pleiotropy and non-overlapping populations), utilities (e.g. GUI), modernization of the software stack (e.g. from py2 to py3), and runtime.

*Some methodological choices are not clearly explained, i.e. how does continuous age of individuals vs discrete time steps interact here;*

This is an important observation. We now clarify that indeed AEGIS considers time in discrete steps (stages). Under section 2: “Age. An integer value, expressed in the time units of simulation steps.”

*how are mutating genes selected. Are they all mutated at every step, sampled independently?*

To address this point, we adjusted wording, from “Mutation rate refers to the probability of each genomic bit to flip” to “Mutation rate determines the independent probability of each genomic bit to flip”.

The Mutation logic is explained under SUPPLEMENTARY INFORMATION > Notes on model design > Mutation rate. In short, mutations occur during reproduction only (there are no somatic mutations), during which each bit has some independent probability of flipping to a different state. The probability is the same for all bits. The probability can differ depending on the direction of the flip (0>1, 1>0).

*p.11 parallelization role is not clear here since, for example, generating the t+1 population from t without modifying t would give the same effect.*

To address this point, we adjusted wording from “This ensures that one individual's life history outcomes do not influence another’s within the same simulation step.” to more neutral “This removes the impact of one individual's life history outcomes on another’s within the same simulation step.”

Indeed, the parallel model choice is not inherently superior to the sequential model.

The main benefit of parallelization are gains in computational efficiency. Instead of iterating over individuals (computing survival, reproduction, etc. of one individual after another), AEGIS iterates over life history traits (survival, reproduction, etc.) and computes life history outcomes (survival / death, offspring / no offspring, etc.) for all individuals in parallel.

Iterating over individuals modifies the population. Hence, the order in which individuals are processed matters. In AEGIS, the order of individuals does not exist, since computation is vectorized, thus there is no inherent order.

(We provide more details under Computation order > Order of processes and Computation order > Order of individuals.)

*In addition, p.12 the description of interactions “modifying the pool of available mates for other individuals” is hard to comprehend if the model is truly parallelized.*

This is an important point. For clarity, though reproduction is parallelized, interaction still occurs between individuals during mate pairing because individuals can mate only (at most) once in each simulation step. Paring among two mating individuals in any given time stage makes them unavailable for mating to the rest of the mating pool. We have modified the test to make this point clearer.

*I really appreciated the clever implementation of the isolation of an evolved population after burn-in for testing the impact of specific parameters, although this limits the study of evolutionary scenarios on stationary populations only. However, this limits the interpretation of parameters’ role to the context of stationary populations only. I know that this is a general assumption of population genetics but numeric simulations should be able to give us insights that are not or less accessible due to assumptions made for mathematical modeling. The authors could maybe show the parameters compared with and without burn-in on models for which they know the expected outcome.*

A burn-in phase is still highly recommended when studying non-stationary populations, maybe even more so than when studying long-term evolutionary equilibria. Since studying evolutionary dynamics entails studying the change of the phenotype, it matters very much what the initial state is, against which the change is occurring.

We would like to draw attention to two results in Figure S6 that illustrate interesting population dynamics.

First, the experiment underlying Figure S6a exhibits a transient evolutionary phenomenon in which the population transitions from low to high survivorship in old age and subsequently returns to low survivorship. This contrasts with the experiment shown in Figure S6d, where the evolutionary trajectory is monotonic over time, characterized by a consistent decline in survivorship across all ages. Together, these results demonstrate that AEGIS is capable of capturing non-linear evolutionary trajectories within the phenotypic landscape.

Second, a comparison of Figures S6a and S6d suggests that the evolutionary loss of survivorship occurs more slowly than its gain. This is evidenced by the continued decline in survivorship beyond the 2M simulation step mark in Figure S6d, whereas survivorship in Figure S6a has already stabilized by that point. This observation indicates that AEGIS can be used to investigate evolutionary trajectories and transient dynamics, rather than being limited to the analysis of equilibrium states.

*Implementing randomization of relevant processes during each step might give significant (see comments from the authors page 10) improvement of outcomes relevance. i.e. averaging results between sims obtained with same parameters but infection / predation inverted.*

We greatly appreciate the thoughtful comment. Indeed, the ability to run the simulations with a different operation order could enhance the robustness of results; qualitatively and quantitatively. Unfortunately, the current version does not provide such an option, though it is planned as an improvement going forward.

*Added to discussion: “Lastly, expanding customization, particularly with regard to modifying the order of computational processes, would further help users test simulation assumptions and improve result robustness.”*

*More generally, as stated by the authors lines 340-343, “not all simulations in AEGIS will necessarily exhibit a flat pre-reproductive mortality” implies that drawing conclusions from simulations should require the implementation of averaged outcomes.*

Discussion around flat or increasing/decreasing pre-reproductive mortality is primarily about long-term equilibrium states; not transient deviations, though such do occur and are indeed best handled by running simulation replicas.

*Definition of intrinsic mortality define as “truly independent of external factors”*

*Starvation mortality being independent of genetics is a strong choice.*

AEGIS framework has been constructed in a way that allows introducing dependence of traits on genetics. In the current version, for sake of simplicity, starvation is independent of genetics. Should there be need for the extension, AEGIS will be able to accommodate it.

*Why only one progeny / reproduction cycle?*

Multiple offsprings can be generated in a reproduction cycle, the maximum number is capped by the user-set parameter MAX_OFFSPRING_NUMBER which is by default 1. The feature is not presented in the paper as it is more advanced, and for many modeling purposes, it is not necessary.

*I regret that there is no further discussion of the final result presented as an example in Fig S.6*

We consider Figure S.6 as a technical test for the constraints provided by initial conditions – in both survival and reproduction – on asymptotic convergence towards equilibrium, rather than an “experiment” per se. Our conclusion for this test is that, given enough runtime, AEGIS is robust to initialization from different states. We now explicitly clarify in the manuscript that Figure S6 serves as a technical convergence test rather than an experimental result, and we briefly discuss the implication of the observed convergence for model robustness. We thank once again the reviewer for helping make our point clearer.

*Discussion starts by mentioning the lack of comprehensive tools, which is not true as indicated by the list of existing solutions mentioned by the authors in introduction. Even though they do not have a GUI, they have clear publications backing them.*

We agree that several well-established modeling frameworks for life history evolution exist and are supported by extensive literature. We have therefore softened the opening of the Discussion to emphasize that AEGIS complements these approaches rather than filling an absolute gap.

*Mentions to late-life plateau argument page 16 and the relative convergence with the Rose experiment are hard to judge since the late-life mortality plateau is debated – and using the model to propose explanations would have been interesting – and the Rose selection shows a relatively low impact on the lifespan. Comparing the outcome with other models while showing the value added by AEGIS on interpretability would have been more relevant.*

We thank the reviewer for this comment and agree that the existence and interpretation of late-life mortality plateaus remain debated. In our simulations, late-life plateaus can emerge, but we do not aim to draw strong biological conclusions from this observation. Rather, this example is intended to illustrate how AEGIS can be used to numerically implement and test established experimental frameworks, such as the Rose selection experiment, and to assess whether their qualitative outcomes are consistent with theoretical expectations from evolutionary biology of aging. The value of this analysis lies in demonstrating that AEGIS can reproduce a well-known experimental paradigm in a transparent and flexible manner, providing a platform for further customization, comparison with alternative models, and more detailed future analyses.

*p. 23 lines 479-481 is an overstatement as 1) numeric simulations only provide empirical testing of hypothesis that will never replace formal analysis of models and 2) it gives an illusion of simplicity where, the large number of parameters make the model difficult to interpret even though the authors implemented a method for isolating a stationary population to play with parameters on.*

We thank the reviewer for this comment, which we gladly embrace. We added the following refinement after the addressed statement: “At the same time, agent-based models like AEGIS require careful interpretation, as they do not offer the same formal analysis as traditional models. While analytical approaches rely on mathematical formalism, agent-based models must use empirical validation through controls, replicated simulations, and statistical analysis. Thus, while AEGIS provides valuable insights, its results must be carefully contextualized through rigorous model design and validation.”

*Reviewer #2:*

*1. The authors choose for parallel execution so that individuals execute their actions independently of another individuals as opposed to have one individual executing after the other. I guess there are context where it makes sense to have both possibilities open, as they are both realistic in different scenarios.*

We thank the reviewer for this consideration. We believe that the parallel computation in AEGIS is one of important factors that greatly improve performance, thus enabling use of several of the features implemented in the simulation. Adding the possibility of sequential computation requires implementation of complex performance boosting techniques. Thus, we defer it to the scope of future improvements.

*2. Mortality/fertility appear to be specified as either an age-independent constant (this gives you a hold over potentially infinite ages) or a vector of age-specific values (this is limited to ages as numerous as the vector length). It would be ideal to have the possibility to have mortality/fertility functions that allow you to specify a different value for any age (possibly infinite)*

We thank the reviewer for raising this point. Allowing mortality and fertility to be defined as age-continuous functions would indeed be a meaningful extension of the framework. Implementing such functionality would require a structural shift from a discrete, numerical representation of life-history traits to a parametric one, which is currently not implemented in AEGIS. While this would allow extrapolation to arbitrarily large ages and the imposition of specific functional forms (e.g. Gompertz mortality), it would also introduce stronger a priori constraints on trait shape.

In the current implementation, life-history traits are represented as age-specific vectors with a user-defined maximum age. This choice was made deliberately to prioritize flexibility and emergent dynamics over imposed functional assumptions. Importantly, this does not constitute a practical limitation for the scenarios explored here: the maximum age and age resolution are user-configurable, allowing the discretization to be refined as needed. In practice, a sufficiently fine age discretization captures smooth age-dependent patterns without imposing a specific parametric form.

Parametric representations may be advantageous when the goal is to enforce empirically observed shapes or test specific functional hypotheses. By contrast, AEGIS was designed to minimize baked-in assumptions about life-history trajectories and instead allow mortality and fertility profiles to evolve freely from genetic, ecological, and demographic interactions.

*3. The authors note that (lines 330-40) that in their simulations aging starts with sexual reproduction. Please note that this replicates a theoretical result by Hamilton 1966.*

We’re excited the reviewer noticed this. Indeed, consistent with the theory, AEGIS evolves age-dependent decrease in survivorship and reproduction right after the onset of sexual maturation. As we point out in the text, AEGIS is not “programmed” for aging to evolve after the onset of sexual maturation; but rather this aspect is entirely emergent.

*4. A major factor that can influence the analysis of life histories as emerging from individual trajectories is demographic heterogeneity, i.e., different subsets of individuals follow different life histories. I find unclear if and how AEGIS deals with demographic heterogeneity when reporting the simulation results.*

We fully agree with the reviewer that demographic heterogeneity is a major determinant of life-history variability and evolution in nature. In AEGIS, heterogeneity is explicitly represented and reported at the individual level: each individual carries a heritable genome (bit string) that maps to age-specific intrinsic mortality and fertility schedules (vectors), and stochastic survival/reproduction outcomes generate realized variation in lifespan and lifetime reproductive success. Consequently, within-population heterogeneity emerges naturally as standing genetic variation plus stochasticity, and is directly visible in the distribution of individual intrinsic life-history trajectories (Figure S3). Importantly, this is not a complication layered on top of the model; because AEGIS is individual-based, heterogeneity is the substrate for selection. Evolutionary change arises from differential survival and reproduction among heterogeneous individuals, rather than from shifts in a population mean alone.

*5. I would like to have a more detailed description of how environmental variability is introduced. Can one for example implement a Markov chain that specifies the environmental state? It seems the key implementable process is some logistic growth (carrying capacity, K).*

We thank the reviewer for this suggestion and agree that explicit environmental state dynamics (e.g., Markov switching between discrete states) can be a powerful way to model environmental variability. In the current version of AEGIS, environmental variability is introduced through (i) resource limitation via carrying capacity and the resulting starvation mortality (Section 1.5), (ii) optional periodic/exogenous abiotic mortality (Methods; illustrated in Fig. 5c and Fig. S4e–f), and (iii) the optional “environmental drift” module, which introduces gradual long-term shifts in environmental conditions (e.g., climate-change–like trends). These mechanisms affect demography indirectly by modulating extrinsic mortality and thus population size and age structure. At present, AEGIS does not implement a general discrete-state environment specified as a Markov chain; however, such state-switching dynamics could be incorporated in future releases given the modular structure of the environmental processes.

*Typos:*

*Line 75: “assumed and built into the.”*

Corrected to “assumed and built into the model.”
